# Comparison of two resilient attachment systems for implant-/mucosa-supported overdentures with a PEKK framework: a clinical pilot study

**DOI:** 10.1007/s00784-021-04342-4

**Published:** 2021-12-21

**Authors:** Istabrak Dörsam, Anastasia Hombach, Christoph Bourauel, Helmut Stark

**Affiliations:** 1 Department of Prosthetic Dentistry, Preclinical Education and Materials Science, Dental School, Rheinische Friedrich-Wilhelms University, Welschnonnenstr. 17, 53111 Bonn, Germany; 2 Oral Technology, Rheinische Friedrich-Wilhelms University, Welschnonnenstr. 17, 53111 Bonn, Germany; 3 Department of Oral Surgery, Dental School, University of Bonn, Bonn, Germany

**Keywords:** Attachment system, Wear, Plaque accumulation, PEKK, Overdenture

## Abstract

**Objectives:**

The aim of the study was to determine differences between Locator and CM LOC attachment systems regarding patient satisfaction and wear of the abutments and their inserts. Plaque accumulation onto the polyetherketoneketone (PEKK) framework and polymethylmethacrylate (PMMA) was investigated for the implant-supported overdentures.

**Methods:**

Seventeen edentulous patients were randomised to receive either Locator or CM LOC system for the first year. The total number of implants was 53. After the randomisation, 25 implants received Locator system, and 28 implants received CM LOC system in the first year. After a period of 12 months, the attachment system was exchanged from either Locator to CM LOC or vice versa. Oral Health Impact Profile (OHIP-14) questionnaires were used to evaluate patient satisfaction, chewing comfort, and pressure lesions. Prosthesis hygiene on the PMMA and PEKK surfaces was evaluated by using Stark plaque index. After the exchange of the abutments, they were stored until the end of the 24 months, and the surface wear of the abutments was analysed using a scanning electron microscope.

**Results:**

Three patients (10 implants) died shortly before the end of the first year. Two patients (7 implants) received only Locator system since CM LOC was not indictable for their implant system. Patient’s satisfaction was increased when the attachment system was changed from Locator to CM LOC after 12 months of wearing time. Chewing ability and comfort were increased when the attachment system was changed from CM LOC to Locator after 12-month wearing time. There was no influence of the change of the attachment system on pressure lesions. The observed plaque accumulation was higher on the PMMA than on the PEKK surface. For the 8 investigated Locator abutments, the wear was within low and middle level. For the 28 investigated CM LOC abutments, the wear was within middle and high level for the terminal implants and between low and middle for the central implants (for patients who received 4 implants).

**Conclusions:**

Patient’s satisfaction and wearing comfort can be improved with implant-supported overdentures with CM LOC abutments in comparison to Locator. There was no clear difference between both attachment systems concerning the chewing ability of the patients. Plaque accumulation was observed on both attachment systems in different areas. Plaque accumulation on PEKK surface was less than on PMMA surface.

**Clinical relevance:**

The CM LOC attachment system offers stable and comfortable wearing conditions for implant-supported overdentures. The use of PEKK as a framework material could reduce the incidence of pressure lesions.

## Introduction

Continuous bone resorption and remodelling processes of the residual ridge complicate the stability and retention of a complete denture. These anatomical problems for patients with atrophied lower or upper alveolar ridge can be solved by implant-supported overdentures. The McGill consensus statement asserts that the minimum choice of treatment for edentulous mandibles is two implants to support an overdenture [1]. Implants provide support, improve retention and stability of overdentures, and reduce or eliminate pressure lesions during chewing and speaking in comparison to complete dentures [2, 3]. Various attachment systems are available for implant-supported overdentures [4]. Overdentures may be retained using splinting attachments (bars and clips) or non-splinting attachments (balls, magnets, telescopic crowns, and resilient attachments). The main disadvantage of splinting attachments is the difficulties to maintain good oral hygiene of the implants and overdenture over a long period of time.

The Locator system was introduced in 2001as a self-aligning system and has double retention in different colours with different resiliencies. Locator abutments are available in different vertical heights; they are loadable, retentive, durable, and have some built-in angulation compensation. Furthermore, repair and replacement are quick and easy.

Zou et al. (2013) [5] compared telescopic crown, bar, and Locator attachments used in removable four implant-supported overdentures for patients with edentulous maxillae regarding implant survival and success rates, biological and mechanical complications, prosthodontic maintenance efforts, and patient satisfaction in a follow-up period of 3 years. It was concluded that Locator system produced superior clinical results compared to telescopic crown and bar attachments in terms of peri-implant hygiene, the frequency of prosthodontic maintenance measures, cost, and ease of denture preparation. However, the long-term complications and comparison to other resilient attachment systems are still not well investigated in clinical studies.

The hybrid anchoring system CM LOC (Cendres + Métaux, Biel/Bienne, Switzerland) is a new resilient attachment system. The main differences of the CM LOC in comparison to the Locator are: No central retention hole for better clinical functionality, increased wearing comfort, and better oral hygiene. Furthermore, this system includes retention inserts made of polyetherketoneketone (PEKK) with four loading levels instead of Nylon inserts for the Locator system.

Polymethylmethacrylate (PMMA) resin is the material of choice for 95% of all total and partial dentures, as it is easy to process, repair, and polish, offers low cost and good physico-chemical properties and acceptable aesthetics [6–8]. For more than 80 years, complete dentures have been made of PMMA resin using various processing methods. Surface characteristics of PMMA dentures, such as roughness, hardness, and wettability, have been reported to be key players in denture-associated stomatitis [9–12].

Dental prostheses need to have smooth surfaces to minimise the retention of plaque and microorganisms [9, 10]. To decrease the accumulation and colonisation of microorganisms, the surface roughness of dental prostheses should not exceed a threshold of 0.2 µm [9, 13]. This can be achieved by common laboratory and chairside finishing and polishing procedures [9, 14].

Polyaryletherketones are high-performance thermoplastic polymers consisting of: polyetheretherketone (PEEK) and PEKK. PEKK has been used as an alternative material for dental frameworks of partially removable dentures, frameworks of partially fixed dentures, and implant abutments [15, 16]. It has 80% higher compressive strength and better long-term fatigue properties than unreinforced PEEK. PEKK has been used as a framework material due to its low weight and compatibility with various veneering materials. Although it is gaining popularity due to its versatility in manufacturing (it can be milled or hot-pressed), only few reports are available for the clinical indications of PEEK. A weak point of the mentioned material is its high rigidity, especially with implants. Such situations might arise causing high mechanical stresses in the material and possible fracture.

The commonly used method to analyse the impact of dental treatments on quality of life and satisfaction of patients is the OHIP, which is a disease-specific measure of an individual’s perception of the social impact of oral disorders on their well-being [17]. Hence, the aim of the present study was to determine the differences with respect to patient satisfaction regarding the attachment system using OHIP-14 questionnaire. The plaque accumulation onto the PEKK framework and PMMA was investigated for the implant-supported overdentures. Moreover, the wear of the abutments was investigated using a scanning electron microscope.

## Material and methods

**Fig. 1:**
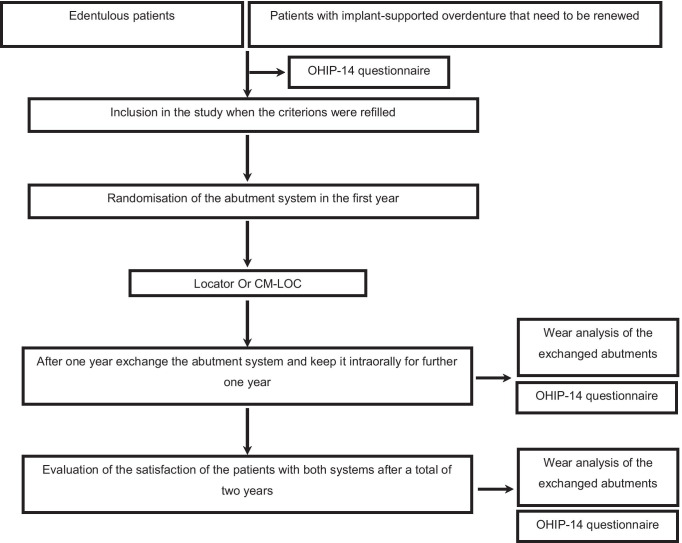
Flowchart of the study protocol

A randomised prospective clinical trial was performed for this study (Fig. 1). The participants were recruited from the pre-examined patient inventory of the Centre for Oral and Maxillofacial Medicine of the University Hospital of Bonn. All participants were recruited from the Department of Oral Surgery, the Department of Oral, Maxillofacial and Plastic Surgery as well as the Department of Dental Prosthetics, Propaedeutics and Materials Science. The recruiting period was 12 months.

The indication for the insertion of two to four implants in the edentulous jaw was defined as a requirement for the participation in the study for improving the retention and stability of the overdentures. An approval was obtained from the ethics committee of the University Hospital of Bonn, Germany (Lfd.Nr. 145/15). A written informed consent was obtained from each participant before starting the treatments.

A total of 17 partially or completely edentulous patients were participated in the study. The mean age of the patients in the study was 73 years. All patients were suffering from poor retention of either complete denture because of the atrophied lower jaw or an existing implant-supported overdenture that was in need to be reconstructed. The patients’ cohort participating in the study consisted of 8 men and 9 women.

Fifteen patients received the implants in the lower jaw and 3 of them in the upper jaw. One patient had received an implant-supported denture in both upper and lower jaws. Table 1 illustrates the position of the overdenture and the number of the supporting implants. The total number of the implants was 53.

**Table 1:** Position of the overdenture, number, and position of the supporting implants for the patients involved in the study

Patients	Position of the overdenture	Number of implants	Position of the implants	Implant system
1	Lower jaw	4	34, 33, 43, 44	Straumann RN
2	Lower jaw	4	34, 32, 42, 44	Straumann RN
3	Lower jaw	4	34, 32, 42, 44	Straumann BL
4	Lower jaw	4	34, 32, 42, 44	Straumann BL
5	Lower jaw	4	34, 32, 42, 44	Straumann RN/NCC
6	Lower jaw	4	34, 32, 42, 44	Straumann RN/NCC
7	Lower jaw	4	33, 32, 42, 43	Xive Densply
8	Lower jaw	4	34, 32, 42, 44	Brånemark
9	Lower jaw	3	42, 32, 34	Brånemark
10	Lower jaw	2	33, 43	Straumann RN
11	Lower jaw	2	33, 43	Straumann RN
12	Lower jaw	2	33, 43	Straumann BL
13	Lower jaw	2	33, 43	Straumann NCC
14	Lower jaw	2	33, 43	Astra Implants
15	Upper jaw	4	23, 22, 13, 12	Straumann RN
16	Upper jaw	4	14, 12, 22, 24	Straumann NCC
17	Upper jaw	3	16, 15, 22	Biomed

Three patients (9 implants) had implant systems that were not compatible to the CM LOC system and thus were only taken into consideration for the evaluation of OHIP questionnaire and plaque index. Three patients died during the time (10 implants) of the clinical trial, and their system could not be completely examined regarding the wear after 2 years.

**Fig. 2:**
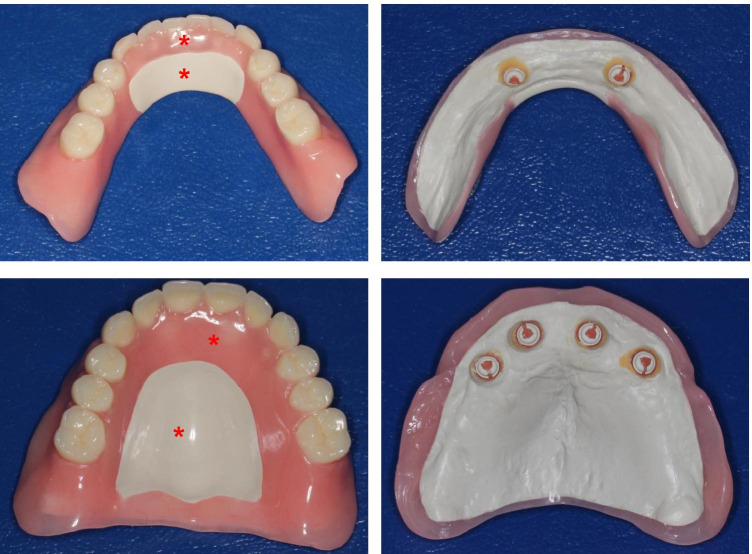
Upper and lower overdentures with PEKK framework and PMMA facing. The star indicates the Position, where the plaque accumulation was taken for the evaluation of prosthesis plaque index

All implant-supported overdentures were made of PEKK framework and PMMA facing (Fig. 1). The layer thickness of both materials in the manufactured overdentures was 2 mm. No PMMA facing was added in the anterior region from the lingual side for the lower overdentures and at the palate region for the upper overdentures. This was considered for the evaluation of the plaque accumulation onto both materials (PEKK and PMMA) using Stark plaque index [18] (Fig. 2). There was direct contact of the PEKK framework to the mucosa and no PMMA relining at the denture base (Fig. 2).

Before the start of the prosthetic treatment, the patients were randomised according to the attachment system during the first year (either Locator or CM LOC as a first system).

After the randomisation, 25 implants received Locator system and 28 implants received CM LOC system in the first year. The selection of the gingival height for the attachment system was based on the clinical requirement, i.e., according to the available vertical dimension of the residual ridge and the insertion depth of the implants. However, the gingival height for both systems was identical per patient and implant position.

Prior to prosthetic treatment, the patients were interviewed using an OHIP-14 questionnaire to assess previous care that patients had received. During the recall visits in a 3-month interval, the patients were asked to fill the OHIP-14 questionnaire again. The plaque accumulation on the PMMA and PEKK surfaces of the overdentures was documented in each recall as well.

The recall visits included professional cleaning and polishing of overdentures and attachment system and pocket flushing around teeth/ implants with chlorhexidine as well.

Three categories were considered for the evaluation of OHIP questionnaire:

1. Patient’s satisfaction and wear comfort (question 1–4).
2. Chewing ability and comfort (question 5–12).
3. Pressure lesions (question 13–14).

These three categories were evaluated with respect to the currently inserted attachment system and to time as well. Each question was answered with a number between 0 and 4 (Table 2). The higher the value, the worse was the patient’s chewing comfort and prosthesis fit, and the higher was the perceived stress during daily activities, such as speaking and chewing.

**Table 2:** The three categories for the evaluation of OHIP questionnaire

Question No	OHIP-14 questionnaire
	**Have you had any problems with your teeth, mouth or dentures in the past month?**
**1**	Having difficulty pronouncing certain words?
**2**	The feeling that your sense of taste was affected?
**3**	The impression that your life in general was less satisfying
**4**	Difficulties zu relax?
	**Has it happened in the past month because of problems with your teeth, mouth or dentures?**
**5**	Did you felt tensed up?
**6**	Did you have to interrupt your meals?
**7**	Were you uncomfortable eating certain foods?
**8**	Have you been rather irritable towards other people?
**9**	Did you find it difficult to perform your daily activities
**10**	Were you completely unable to do anything?
**11**	Did you feel a little embarrassed?
**12**	Has your nutrition been dissatisfying?
	**Last month, did you have.**
**13**	Pain in the Oral area?
**14**	A feeling of uncertainty about your teeth, your mouth or your denture?
	**OHIP_14 Code**
	**very often = 4, often = 3, sometimes = 2, hardly = 1, never = 0**

**Fig. 3:**
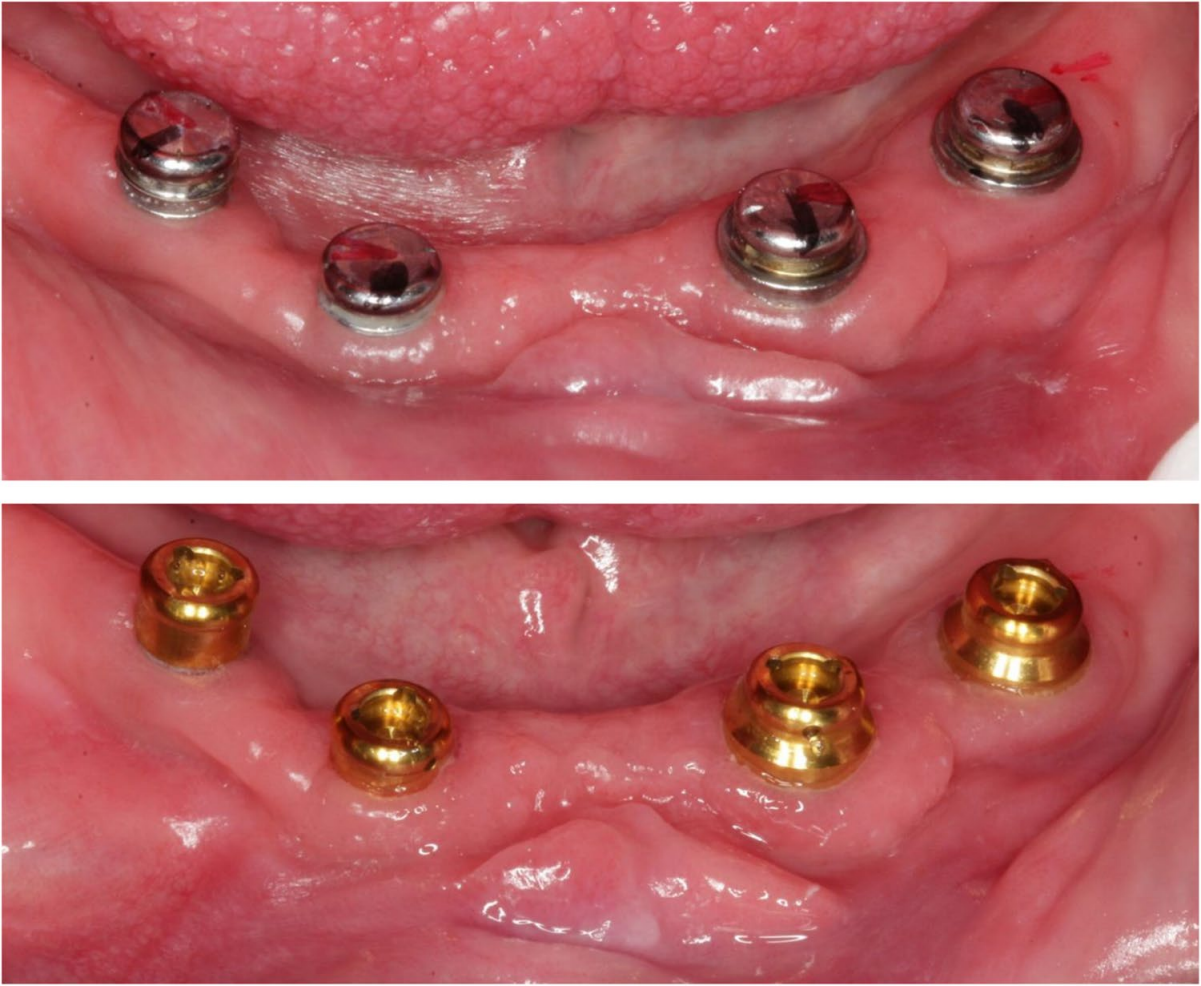
Exchange of the attachment system from CM LOC (upper photo) to Locator (lower photo) after 12 months

After 12 months, the anchoring system was exchanged from either Locator to CM LOC or vice versa to record comparable periods of adjustment (Fig. 3). During the exchange appointment, a relining of the denture base was considered as well.

The exchanged abutments were cleaned with alcohol in an ultrasonic bath and finally sterilised. Subsequently, a gold-platinum plating was performed using a sputtering device. The surfaces of the abutments were analysed using a scanning electron microscope (Philipps XL 30, FEI Electron Optics B.V., Eindhoven, Netherlands). The images of each abutment were examined in different magnifications (100 × , 200 × , 500 × , 1000 ×). The wear was classified during the analysis in low, middle, and high level of wear. After the end of the second period (i.e. after 24 months), patients were given the option to decide to keep their favoured anchoring system.

Three patients (10 implants) died shortly before the end of the first year. Two patients (7 implants) received only Locator system since CM LOC was not indictable for their implant system. For this reason, only 8 Locator abutments were available for the wear analysis. For the CM LOC system, 28 abutments were available.

## Results

### Evaluation of OHIP questionnaire

**Fig. 4:**
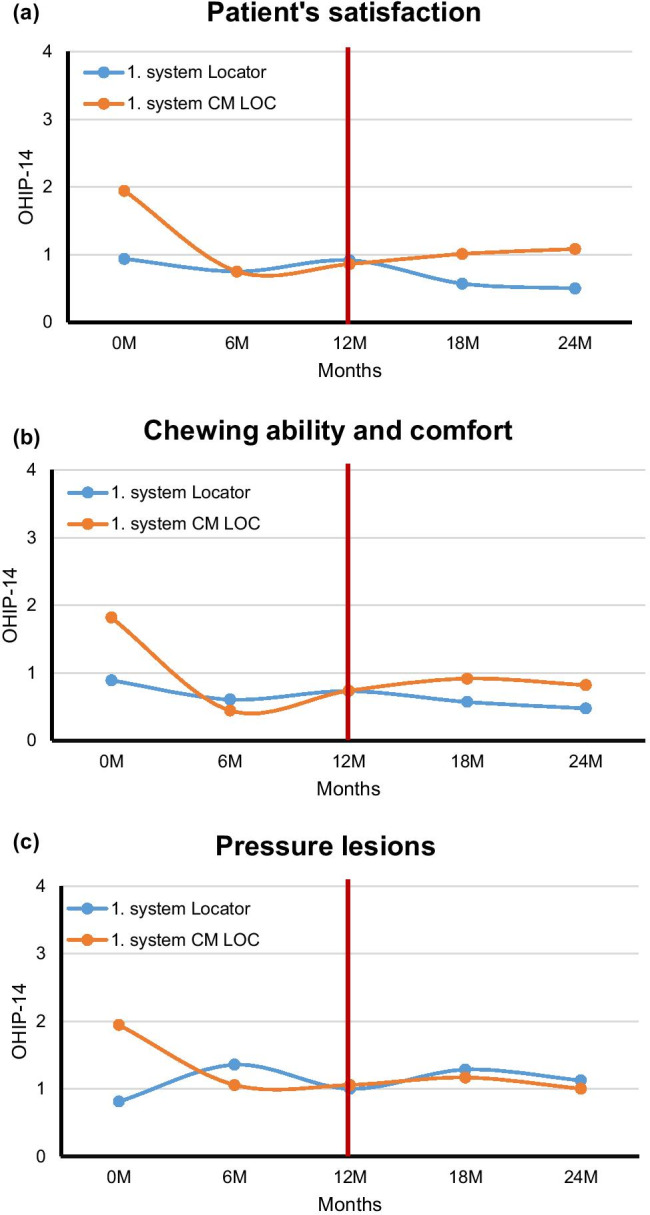
(**a**) Patient’s satisfaction, (**b**) and chewing ability and comfort, and (**c**) pressure lesions at the initial condition and after 6, 12, 18, and 24 months of receiving implant-supported overdenture

Independent on the attachment system, patient’s satisfaction and wear comfort as well as chewing ability and comfort were improved after receiving implant-supported overdenture in comparison to the initial condition (Fig. 4a–b). However, the above mentioned two categories were highly improved with four supporting implants instead of two implants in the lower jaw. Pressure lesions were reduced after receiving implant-supported overdenture in comparison to the initial condition (Fig. 4c).

Results regarding the influence of the attachment system on the evaluation categories of the OHIP questionnaire were as follows:

Patient’s satisfaction was increased when the attachment system was changed from Locator to CM LOC after 12 months of wearing time. When CM LOC was used as a first system, there was no noticeable change in the patient’s satisfaction after changing the system to Locator (Fig. 4a).

Chewing ability and comfort were increased when the attachment system was changed from CM LOC to Locator after 12 months of wearing time. There was no noticeable change in the chewing ability and comfort after changing the system from Locator to CM LOC (Fig. 4b). There was no influence of the change of the attachment system on the pressure lesions (Fig. 4c).

The patients at the end of the 2 years decided to keep the second system that they received, i.e., 8 patients kept the Locator system, and 4 patients kept the CM LOC system (the three deceased patient had CM LOC as a second system). In general, the patients felt more comfortable and more secure that the denture was in the final position with the CM LOC system. One reason is the fact that the PEKK inserts are stiffer than nylon inserts and produce a hearable click upon insertion of the denture in the mouth.

### Plaque accumulation on the overdenture and abutments

**Fig. 5:**
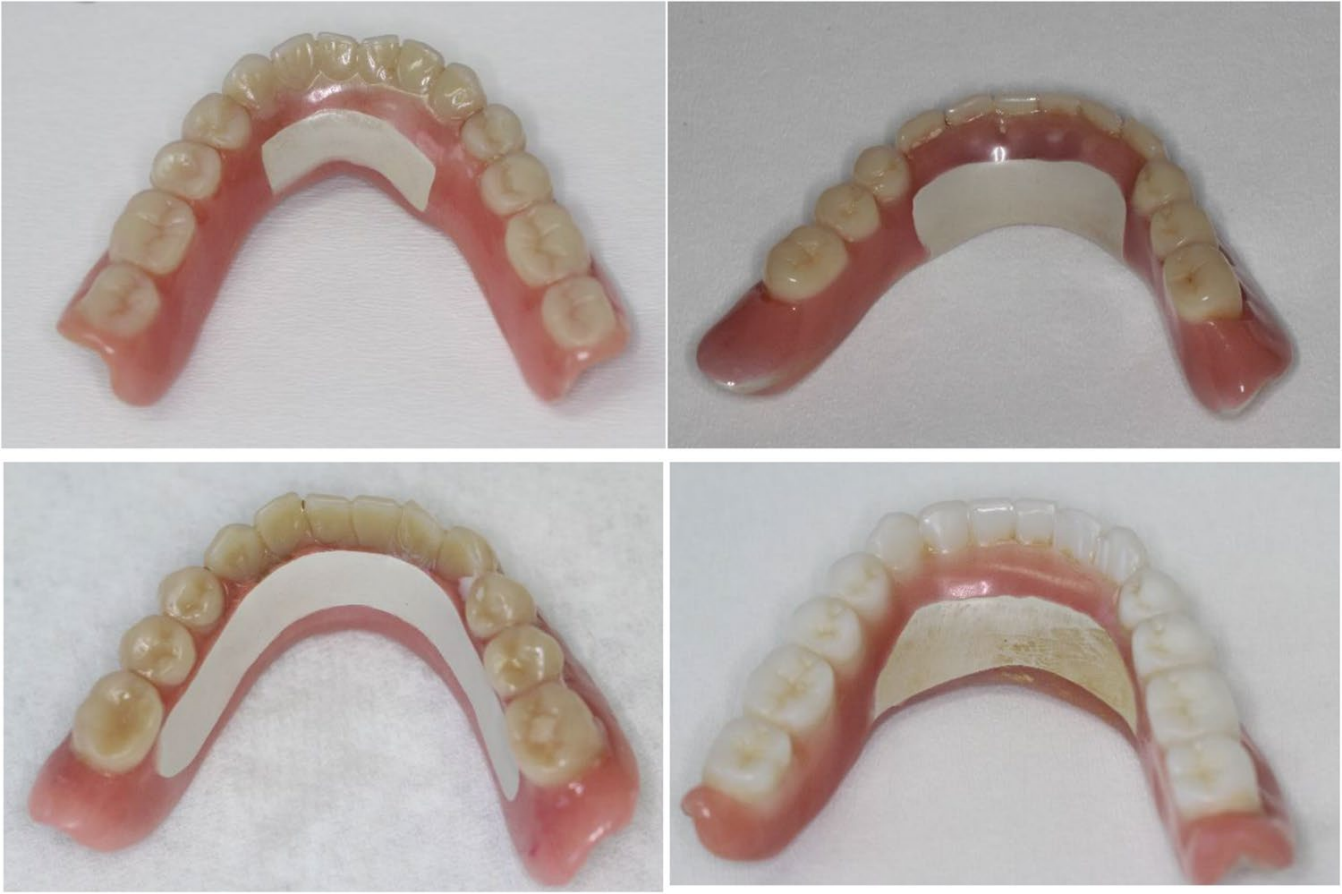
Examples of the prosthesis’s hygiene and plaque accumulation after 24 months of wearing the prosthesis without any professional cleaning and polishing during this period

With respect to plaque accumulation on PMMA and PEKK surfaces, the observed plaque accumulation was higher on the PMMA than on the PEKK surface. However, the magnitude of the plaque accumulation regardless of the surface material was strongly depending on the general oral hygiene and cleaning ability of the overdenture (Fig. 5).

**Fig. 6:**
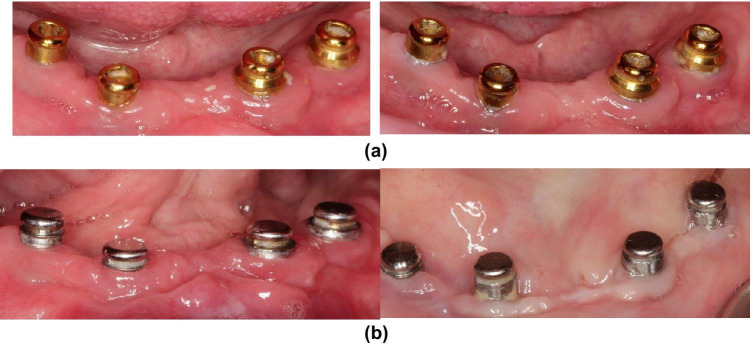
(**a**) Regions of plaque accumulation on the Locator abutments. (**b**) Regions of plaque accumulation on the CM LOC abutments

The typical region for plaque accumulation on the Locator was the central retention hole and the lingual/interproximal surface which are not easy reachable for the patients to clean (Fig. 6a). For the CM LOC system, the typical plaque accumulation region was the horizontal groove of the abutment on the lingual/interproximal surface (Fig. 6b).

During the 2 years, no peri-implantitis was observed. The probing depth was maximum 4 mm. There was no fractures of the overdentures, only relining. The frequency of relining was 6–12 months.

### Wear of the abutments

Eight Locator abutments and 28 CM LOC abutments were available for the wear analysis.

For the CM LOC, it was possible to localise the position of the worn surface by using the available notch at the top of the abutment. On the contrary, it was not possible to localise the exact position of the wear on the surface of the Locator (Table 3).

**Table 3:** Wear regions of the Locator and CM LOC systems after 12 months in mouth

Patients	Position of the implant	Implant system	Diameter of the implant	Gingiva height of the abutment	Level of wear
**Locator system**					
9	32	Brånemark	3.75 mm	3 mm	Low
	42	Brånemark	3.75 mm	2 mm	Middle
	44	Brånemark	3.75 mm	2 mm	Middle
11	33	Straumann RN	4.1 mm	1 mm	Middle
	43	Straumann RN	4.1 mm	1 mm	Middle
15	13	Straumann RN	4.8 mm	1 mm	Middle
	12	Straumann RN	4.8 mm	2 mm	Middle
	22	Straumann RN	4.8 mm	2 mm	Middle
	23	Straumann RN	4.8 mm	2 mm	Middle
**CM LOC system**					
2	34 distal	Straumann RN	4.1 mm	2 mm	Middle
	34 mesial				Middle
	32 lingual		4.1 mm	2 mm	Middle
	32 labial				Middle
	42 mesial		4.1 mm	2 mm	Middle
	42 distal				Middle
	44 distal		4.1 mm	2 mm	Middle
	44 mesial				Middle
3	34 buccal	Straumann BL	4.1 mm	5 mm	Middle
	34 lingual				High
	32 mesial		4.1 mm	4 mm	High
	32 distal				Middle
	42 labial		4.1 mm	3 mm	Middle
	42 lingual				Middle
	44 mesial		4.1 mm	4 mm	High
	44 distal				High
5	34 distal	Straumann RN	4.1 mm	1 mm	Middle
	34 mesial				Middle
	32 mesial	Straumann NCC	3.3 mm	4 mm	Low
	32 labial				Low
	42 labial	Straumann NCC	3.3 mm	4 mm	High
	42 lingual				High
	44 mesial	Straumann RN	4.1 mm	1 mm	Middle
	44 distal				Middle
6	32 lingual	Straumann NCC	3.3 mm	1 mm	Middle
	32 labial				High
	42 distal	Straumann NCC	3.3 mm	1 mm	High
	42 lingual				Middle
	44 lingual	Straumann RN/	4.1 mm	1 mm	Low
	44 labial				Low
	34 mesial	Straumann RN	4.1 mm	1 mm	Middle
	34 buccal				Middle
9	32 labial	Brånemark	3.75 mm	1 mm	Low
	32 lingual			1 mm	Low
	34 lingual		3.75 mm		High
	34 buccal				High
	42 mesial		3.75 mm	2 mm	Middle
	42 distal				Middle
	44 mesial		3.75 mm	1 mm	Middle
	44 distal				Middle
4	34 distal	Straumann BL	4.1 mm	4 mm	Middle
	44 distal		4.8 mm	5 mm	Middle
17	16 distal	Biomed	4.1 mm	0.73 mm	Middle
	16 buccal				Low
	15 mesial	Biomed	4.1 mm	4 mm	Low
	15 palatal				Low
	22 palatal	Biomed	5.0 mm	4 mm	Middle
	22 distal				Middle

**Fig. 7:**
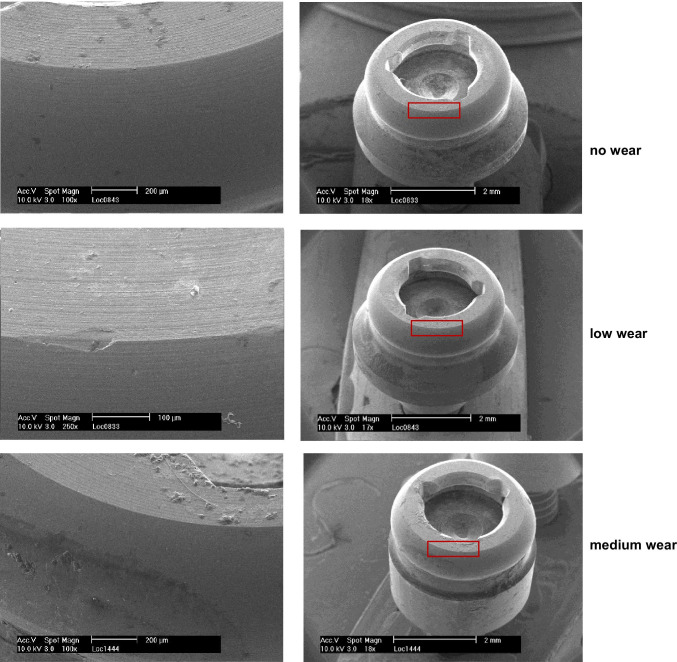
Wear regions of the Locator abutments after 12 months of use intraorally

In general, most of deceased high-level wear was detected for the lower jaw regardless of the type of the attachment system, taking into consideration, that all three upper dentures were constructed as overdenture or with a transversal band. For the 8 investigated Locator abutment, the wear was within low and middle level. The typical region of wear at the Locator surface was at the engaging part above the horizontal groove for the inserts (Fig. 7).

**Fig. 8:**
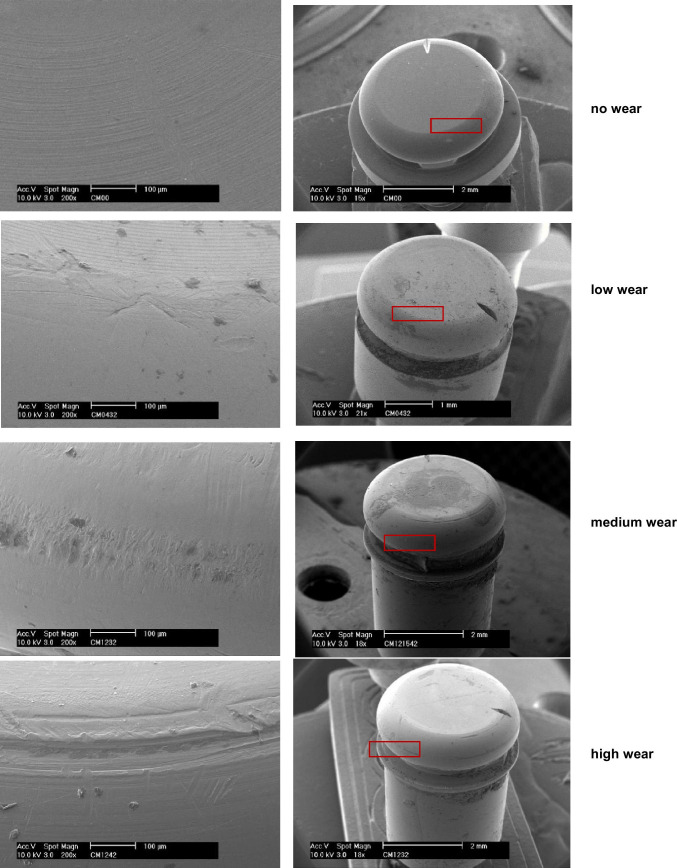
Wear regions of the CM LOC abutments after 12 months of use intraorally

For the 28 investigated CM LOC abutments, the wear was within middle and high level for the terminal implants and between low and middle for the central implants (for patients who received 4 implants). The typical region of wear at the CM LOC surface was similar to the Locator, i.e., at the engaging part above the horizontal groove for the inserts (Fig. 8). It was not possible to find a correlation between the wear pattern/region and the implant system.

## Discussion

The clinical outcome of patient satisfaction and wearing comfort for implant-supported overdentures with Locator and CM LOC attachment systems was investigated in this study. The plaque accumulation on the abutments and on the surface of the dentures was analysed as well. The difference in plaque accumulation between PMMA and PEKK was studied. Finally, the wear behaviour of the Locator and CM LOC attachment systems was compared.

Patients’ satisfaction increased when the attachment system was changed from Locator to CM LOC after 12 months of wearing time. The reason could be that the patients felt more comfortable and more secure that the denture is in the final position with the CM LOC system. This is due to the fact that PEKK inserts are stiffer than those made of nylon and make a clear hearable click by inserting the denture in the mouth. In addition, the need of replacement of PEKK inserts was much lower than nylon inserts of the Locator. Ultimately, this means for the patient less visits to the dentist and lower costs for new inserts.

Chewing ability and comfort slightly improved when the attachment system was changed from CM LOC to Locator after 12-month wearing time. In contrast, there was no noticeable change in the chewing ability and comfort after changing the system from Locator to CM LOC.

There was no influence of the change of the attachment system on the occurrence of pressure lesions. All overdentures constructed with a PEKK framework had a direct contact to the residual ridge, i.e., there was no PMMA facing at the base of the denture. This could have a relation, in addition to the influence of the implant support, to the reduction of the pressure lesion at the free end extensions. During the study period of 24 months, the dentures were only once relined and that was after exchanging the attachment system.

Plaque accumulation is clearly influenced by the quality of denture hygiene. In our study, the plaque accumulation on the PEKK and PMMA surfaces was evaluated for the patients who received implant-supported overdentures. The observed plaque accumulation was higher on the PMMA than on the PEKK surface. This could be related to the difference in the roughness of both materials. The optical observation of the plaque accumulation can be considered as one limitation of this study, since there was no detailed examination of material roughness, polishing degree of the denture surface, and their effect on the plaque adhesion.

The typical region for plaque accumulation with Locator systems was the central retention hole for all the patients. This consequently negatively affected the retention of the inserts, since the overdenture could not be inserted to the final position with the central hole blocked by plaque. For some older patients with motor difficulties, the central hole had to be filled with filling material, and a different nylon insert had to be used. For the CM LOC system, the typical plaque accumulation region was the horizontal groove of the abutment. For most patients, even those with good oral hygiene and brushing ability, it was impossible to remove the plaque. During the recall visits, it was difficult as well to clear this groove with professional instruments. However, retention of the inserts was not negatively affected by the plaque accumulation in this region. For both attachment systems, the lingual/interproximal surfaces were observed as well as typical areas for the plaque accumulation.

In the study of Naguib et al. 2019 [19], the use of PEKK inserts was discussed. The PEKK insert has an oval C-shaped design which provides a slot in the insert. This slot is intended to allow expansion of the insert resulting in a reduced wear of the insert material [20].

The difference of resiliency between the PEKK inserts of CM LOC and the more resilient nylon inserts of the Locator system can be the reason for more wear of the COM LOC abutments. The PEKK material has a higher ratio and sequence of ketone Groups, which increase the rigidity of the polymer chain [20]. PEKK is a less resilient material, which might tend to higher wear due to friction during denture movement [20–22]. In the present study, the wear of the CM LOC abutments was higher than the wear observed for the Locator abutments.

The present study had some limitations: Firstly, the number of the patients and inserted implants was small. This was due to the fact that COM LOC abutment are not compatible to all implant systems. For this reason, several patients were excluded from the study. Consequently, it was not possible to make a statistical analysis with convincing results. Secondly, three patients had implant systems that were not compatible to CM LOC® anchoring system and additional three died during the study. For this reason, the wear analysis of the Locator was restricted to only 11 abutments.

Thirdly, the geometry of both abutment systems was individual depending on the implant design. This made the comparison of the abutment wear difficult.

For possible clinical trials in the future, more patients need to be included in the study, and additional abutment system could be compared, namely locator R-Tx.

## Conclusion

Patient’s satisfaction and wearing comfort can be improved with implant-supported overdenture with CM LOC abutments in comparison to Locator. There was no clear difference between both attachment systems concerning the chewing ability of the patients. Plaque accumulation was observed on both attachment systems in different areas.
